# Structure of the Pseudokinase VRK3 Reveals a Degraded Catalytic Site, a Highly Conserved Kinase Fold, and a Putative Regulatory Binding Site

**DOI:** 10.1016/j.str.2008.10.018

**Published:** 2009-01-14

**Authors:** Eric D. Scheeff, Jeyanthy Eswaran, Gabor Bunkoczi, Stefan Knapp, Gerard Manning

**Affiliations:** 1 Razavi Newman Center for Bioinformatics, Salk Institute for Biological Studies, La Jolla, CA 92037, USA; 2 Structural Genomics Consortium, University of Oxford, Old Road Campus, Roosevelt Drive, Oxford OX3 7DQ, UK; 3 Department of Clinical Pharmacology, University of Oxford, Old Road Campus, Roosevelt Drive, Oxford OX3 7DQ, UK

**Keywords:** PROTEINS, SIGNALING

## Abstract

About 10% of all protein kinases are predicted to be enzymatically inactive pseudokinases, but the structural details of kinase inactivation have remained unclear. We present the first structure of a pseudokinase, VRK3, and that of its closest active relative, VRK2. Profound changes to the active site region underlie the loss of catalytic activity, and VRK3 cannot bind ATP because of residue substitutions in the binding pocket. However, VRK3 still shares striking structural similarity with VRK2, and appears to be locked in a pseudoactive conformation. VRK3 also conserves residue interactions that are surprising in the absence of enzymatic function; these appear to play important architectural roles required for the residual functions of VRK3. Remarkably, VRK3 has an “inverted” pattern of sequence conservation: although the active site is poorly conserved, portions of the molecular surface show very high conservation, suggesting that they form key interactions that explain the evolutionary retention of VRK3.

## Introduction

Protein kinases modulate the activity of most cellular processes by phosphorylation of substrate proteins, leading to changes in activity, localization, and turnover (Hunter, 2000). However, 49 human protein kinase domains are predicted to be enzymatically inactive (pseudokinases), owing to the lack of essential catalytic residues (Manning et al., 2002b). Many pseudokinases are conserved throughout metazoans (Caenepeel et al., 2004; Manning et al., 2002a), indicating that their kinase domains retain essential functions. Known pseudokinase domain functions include coreceptors (ErbB3, EphA10, EphB6, CCK4), regulators of a second active kinase domain in the same protein (the pseudokinase domains of JAKs and GCN2), guanylate cyclases, and scaffolding proteins (STRAD, ILK, KSR, TRRAP, Trb3) (reviewed in Boudeau et al. [2006]). Several pseudokinases have been implicated in cancer and other diseases (Manning and Caenepeel, 2006). A few kinases have been shown to retain activity even after loss of key functional residues, including the WNK family and Titin; their structures have revealed compensatory changes that maintain catalytic function (Mayans et al., 1998; Min et al., 2004). Remarkably, CASK, which was initially predicted to be a pseudokinase based on loss of a key motif (Manning et al., 2002b), was recently shown through structural and biochemical analysis to bind ATP and retain catalytic activity (Mukherjee et al., 2008). However, no “genuine” pseudokinase structure, which truly lacks catalytic activity, has been described to date.

VRK3 is a pseudokinase member of the vaccinia related kinase (VRK) family, which also includes two active vertebrate paralogs (VRK1 and VRK2), one or two orthologs in all metazoans, and an ortholog in most poxviruses, including the founding member, vaccinia virus B1R (Boyle and Traktman, 2004; Manning et al., 2002a, 2002b; Nezu et al., 1997; Nichols and Traktman, 2004; Vega et al., 2003). The VRK family is part of the CK1 kinase group, which displays distinctive alterations to some motifs conserved in almost all other kinases, but still assumes the standard kinase fold (Scheeff and Bourne, 2005).

VRK1 is an active nuclear kinase whose substrates include p53, ATF, Jun, BAF, and histone H3, and is involved in cell cycle, chromatin condensation, and transcriptional regulation (Kang et al., 2007; Lopez-Borges and Lazo, 2000; Nichols and Traktman, 2004; Nichols et al., 2006; Sevilla et al., 2004; Valbuena et al., 2007, 2008; Vega et al., 2004). VRK2 also phosphorylates p53 and BAF (Blanco et al., 2006; Nichols et al., 2006). VRK2 has two splice forms that localize either to the nucleus and cytoplasm or to the ER and mitochondria (Blanco et al., 2006). The alternatively spliced C-terminal tail interacts with and regulates several components of the JNK signalosome (JIP-1, TAK1, and MKK7), and with BHRF1, an Epstein-Barr virus homolog of Bcl-2, independent of kinase activity (Blanco et al., 2007, 2008; Li et al., 2006).

VRK3 is the only VRK to lack enzymatic activity (Nichols and Traktman, 2004), but it is known to inhibit Erk signaling by binding and activating the Erk phosphatase, VHR (Kang and Kim, 2006, 2008). VRK3 is in turn transcriptionally induced by Erk (Kang and Kim, 2006).

We present here the crystal structure of the VRK3 kinase domain, the first solved pseudokinase, along with that of its active paralog, VRK2. We examine the changes that have occurred in VRK3 upon loss of catalytic activity, and the implications of these changes for its remaining functions. We also describe shifts in conservation patterns in VRK3 that suggest a location for a protein binding site, perhaps that of VHR. Finally, we examine the implications of the VRK3 structure for the many pseudokinases for which only sequence information is available.

## Results

### VRK3 and VRK2 Are Structurally Very Similar

**Figure 1:**
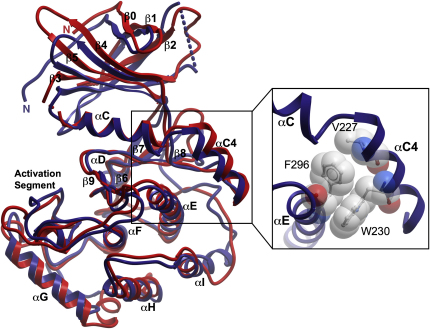
Superimposition of the Kinase Domain Structures of VRK3 (blue) and VRK2 (red) (Inset) Magnified view of highly conserved residues linking αC4 to αE.

The catalytic domain structures of VRK3 (residues 148–472) and VRK2 (residues 15–330) reveal the typical protein kinase fold (Figure 1; see Table S1 available online for crystallographic data). We name elements in the fold according to standard convention, which is based on protein kinase A (PKA) (Bossemeyer et al., 1993). VRK3 is well structured, with only a short loop between β2 and β3 (residues 188–197) not defined by electron density and assumed to be disordered. VRK2 is also well structured, except for the ATP-binding glycine-rich loop (G-loop, G36-G41) and N67 in the loop linking β3 to αC. The unstructured G-loop is commonly seen in kinase structures without bound ATP. In contrast, the VRK3 G-loop is well ordered, with low B-values that indicate that it is rigid in the absence of ATP. Both kinase structures are in an active conformation (Huse and Kuriyan, 2002): the two lobes of the kinase domain are closed, αC is rotated inwards, and a key ion pair between K72-E91^PKA^ is formed. Further, a hydrophobic spine that stabilizes the active conformation (Kornev et al., 2006) is assembled in both structures. Finally, the activation segments of both structures are in conformations compatible with activity (Huse and Kuriyan, 2002; Nolen et al., 2004), and also have low B-values, indicating that they are more rigid than in most kinases. This is consistent with rigid activation segments seen in other CK1-group kinases (Nolen et al., 2004).

The overall structures of VRK3 and VRK2 are similar and superimpose with an RMSD of 2.3 Å, using 291 main-chain positions (Figure 1; see Table S2). Both VRK2 and VRK3 have nearly identical levels of structural similarity to other protein kinases, indicating that the loss of activity in VRK3 has not lead to any major structural change (Table S2). This contrasts greatly to expectations based on sequence alignment: although VRK1 and VRK2 share 56% sequence identity, similar to many vertebrate paralogs, VRK3 is only 38% identical to VRK2, and was previously predicted to be a pseudokinase of degraded structure (Manning et al., 2002b).

Still, there are a number of small differences in secondary structure. In the C-terminal lobe, two helices are shortened in VRK2 relative to VRK3 and CK1: αD is truncated, containing only a half a helical turn, and αI is shortened and loses its distinctive kink (Scheeff and Bourne, 2005). Conversely, VRK3 has a β-hairpin linking αG and αH in place of the short helix seen in VRK2 and CK1. This region is often important in substrate recognition (Bossemeyer et al., 1993; Huse and Kuriyan, 2002), and its modification in VRK3 might reflect its catalytic inactivity.

The most striking unique feature of the VRK structures is an additional helix (αC4) between αC and β4 that anchors αC to the C-terminal lobe (Figure 1). A similar insert is found in the sequences of all VRK family members (Figure S1). Two highly conserved positions in αC4 pack against αE: a conserved hydrophobic residue at V227^VRK3^ and an aromatic residue at W230^VRK3^. Both residues interact with a highly conserved F/Y residue at F296^VRK3^ in helix αE to form the center of a small hydrophobic core linking the two helices (Figure 1). The tight link between αC4 and αE extends the contact region between the two lobes of the kinase, likely inducing the catalytic domain to remain in an active closed conformation (Huse and Kuriyan, 2002) and suggesting that all VRKs are constitutively active. This is supported by the lack of a conserved activation loop phosphorylation site or h**r**D arginine in VRKs (see below; Figure S1). The only other kinases with a known helix insert in this region are SRPKs, but that helix has a very different orientation (Nolen et al., 2001), and the SRPKs are not close relatives of the VRKs (Manning et al., 2002b). Although the SRPK helix appears to be an independent invention, it has been proposed to have a similar function (Nolen et al., 2001).

### VRK3 Contains Multiple Sequence Changes that Lead to Inactivity

**Figure 2:**
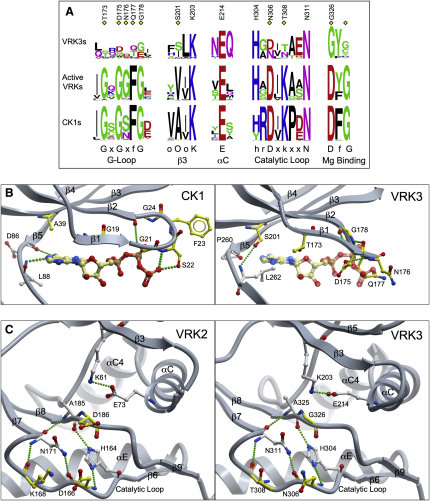
The Altered Active Site Region of VRK3 (A) Sequence conservation logos for key motifs in VRK3s, active VRKs, and the CK1 family, based on appropriate subsets of a curated sequence/structure alignment of diverse homologs (see methods). Standard motifs seen across protein kinases are shown below the logo representation (uppercase residues are conserved nearly to identity across all protein kinases, whereas lowercase residues are partially conserved; x indicates any residue; o, hydrophobic). Numbered VRK3 residues are shown in panels B and C. Yellow diamonds highlight positions in which the VRK3s violate evolutionary constraints seen in active VRKs and CK1s. (B) Comparison of the ATP binding pockets of CK1 and VRK3. ATP from the CK1 structure is modeled into VRK3 based on structural superimposition. Yellow marks VRK3 residue positions that violate evolutionary constraints (as per panel A), whereas white marks other residues with relevant contacts. Homologous CK1 residues are colored identically to VRK3 residues. Hydrogen bonds are shown as green dotted lines. Portions of the structures are not shown to improve clarity. (C) The active site region of VRK3 compared with that of VRK2, labeled as in panel B.

Although the overall fold of VRK3 is intact and similar to VRK2, disruptions to several key motifs render it catalytically inert. The motifs required for kinase catalytic activity have been extensively characterized (Hanks and Hunter, 1995; Kannan et al., 2007; Scheeff and Bourne, 2005) and include a glycine-rich G-loop, a lysine-glutamate ion pair at K72-E91^PKA^, a catalytic loop (hrDxkxxN), and a metal-binding motif (DfG). Some of these motifs are severely degraded in all vertebrate VRK3 orthologs, indicating that the loss of activity occurred early in the evolution of VRK3 (Figure 2A, Figure S1). Here, we systematically examine changes in the catalytic motifs within VRK3, in both a structural and evolutionary context.

### The ATP Binding Site of VRK3 Is Highly Degraded

**Figure 3:**
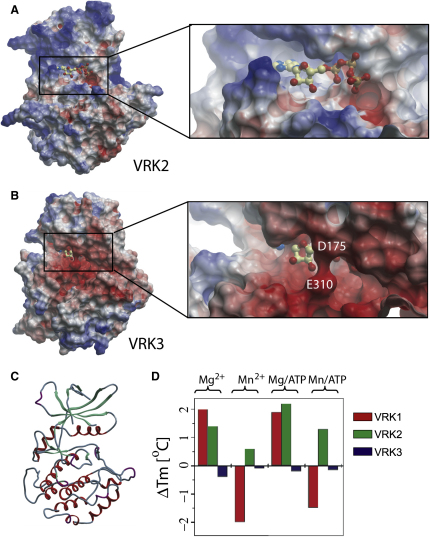
The Nonfunctional ATP Binding Pocket of VRK3 (A) Electrostatic surface view of VRK2. The ATP molecule (ball-and-sticks view) is modeled from CK1. (B) Equivalent view of VRK3 shows highly acidic binding pocket and partial occlusion of modeled ATP. (C) Secondary structure representation of VRK2 showing the orientation of electrostatic representations. (D) ATP binding capability of the VRKs, from a thermal denaturation assay. ΔTm is the shift in melting temperature when purified protein is placed in solution with ligand, and is the average of three experimental measurements (see Experimental Procedures).

The protein kinase G-loop (GxGxfG motif) positions ATP for efficient catalysis by interacting with its phosphate groups. The glycines provide conformational flexibility, enabling hydrogen-bond formation between the backbone of the loop and the γ-phosphate of ATP (Madhusudan et al., 2002). The lack of side chains in glycine also prevents steric clashes with ATP (Bossemeyer, 1994). The loop motif is highly degraded in all VRK3 orthologs, with the glycines often replaced by substantially larger residues (Figures 2A and 2B). We modeled ATP in the VRK3 structure by superimposing the ATP-bound form of CK1, which is the closest relative of the VRKs (Table S2), and has a highly similar loop motif (Figure 2A). This revealed several distinct features that would hinder ATP binding in VRK3 (Figure 2B). The G-loop forms steric clashes with ATP at Q177^VRK3^ and the surrounding backbone. The analogous position in CK1 (S22^CK1^) instead hydrogen bonds to the phosphate, contributing to ATP binding. Interestingly, D175^VRK3^ occupies the ATP binding site, mimicking an ATP phosphate in an ATP bound state. These and other changes produce a highly acidic ATP binding pocket that is likely to repel rather than accept the negatively charged phosphates of ATP (Figure 3). Neither D175^VRK3^ nor Q177^VRK3^ is well conserved (Figure 2A), but the acidic and bulky residue substitutions at these positions in VRK3 orthologs imply inhibition of ATP binding in all VRK3s.

Other changes to the ATP binding pocket occur outside of the G-loop. D86^CK1^ and L88^CK1^ (in the interlobe linker) hydrogen bond with the adenine ring of ATP (Figure 2B). D86^CK1^ accepts a hydrogen bond from ATP via its backbone carbonyl; in VRK3 the equivalent proline (P260^VRK3^) has an altered backbone conformation and can no longer bind ATP. L88^CK1^ donates a hydrogen bond to ATP via its backbone amine, but in VRK3, L262^VRK3^ has shifted conformation such that the side chain would sterically clash with the adenine ring. The backbone hydrogen bond of L262 is instead accepted by the side chain of S201^VRK3^ from β3, a very unusual substitution to a position that is always hydrophobic (Figure 2A). Further, VRK3 has a conserved substitution to a large hydrophobic residue at F313^VRK3^ in β7 of the C-terminal lobe, a position that is conserved as a smaller hydrophobic residue in active VRKs and CK1s (Figure S1, not shown in Figure 2). The combined affect of these changes is to fill in much of the region where the adenine ring would normally bind. The changes also complete a conserved hydrophobic spine seen in all protein kinases that is normally completed by the adenine ring (Kornev et al., 2008), likely allowing VRK3 to be highly stable in the absence of ATP. Taken together, the substantial array of alterations to the ATP binding pocket of VRK3 strongly suggest that it is nonfunctional, and that the structure has evolved adaptations to compensate for the loss of ATP binding.

### VRK3 Does Not Bind ATP or ATP Analogs

We tested the degeneration of the ATP binding pocket by assaying the ability of VRK3 to bind ATP or ATP-analog inhibitors. Purified VRK1, VRK2, and VRK3 proteins were screened against ATP (plus divalent cations) and a kinase-directed library of 605 low-molecular-weight potential inhibitors by monitoring changes in protein melting temperature during thermal denaturation (Lo et al., 2004). This method ranks inhibitors based on an observed shift in melting temperature (Tm), which has been shown to correlate well with the binding strength and IC_50_ values (Bullock et al., 2005). Although VRK1 and VRK2 bound ATP (Figure 3D), as well as several inhibitors (Tables S3 and S4), VRK3 bound neither, confirming that the ATP binding site in VRK3 is nonfunctional. VRK3 was also screened against ATP only (without divalent cations), and was found to also not bind in these conditions (data not shown). In support of the notion that VRK3 is rigid and stable in the absence of ATP, it had the highest native Tm of the three proteins: melting temperatures were 49.8°C, 47.5°C, and 45.8°C for VRK3, VRK2, and VRK1, respectively.

### Most Pseudokinases Have Degraded G-Loop Motifs

The lack of ATP binding in VRK3 suggested that degradation of the G-loop motif might serve as an independent predictor of kinase inactivity, beyond the motifs previously used. We examined this motif in all human kinase domains (Manning et al., 2002b). Almost all active kinases retained the basic GxGxxG motif, whereas a few had conservative substitutions to the first and third glycines, resulting in a generalized motif of (G/S/A)xGxx(G/S/A). Only 16 active kinase domains violate this motif (∼4%), including the four WNKs, which have a known active site modification that changes the motif (Min et al., 2004) (Table 1). However, 41 of the 49 current predicted pseudokinase domains (∼84%) violate the motif, and 28 have severe motif changes, often replacing glycines with acidic residues, as seen in VRK3 (Table S5). This indicates that most pseudokinases have divergent G-loops, and probably either cannot bind ATP or cannot properly orient it for catalysis. Remarkably, CASK and Titin, which have modifications to another key motif yet retain catalytic activity (Mukherjee et al., 2008; Takano-Ohmuro et al., 1992), do not violate the G-loop motif (Table 1).

Examination of the VRK3 structure revealed another important predictor of kinase activity: A39^CK1^ in β3 forms part of the adenine binding pocket and is replaced by S201 in human and many other VRK3s, as described above (Figures 2A and 2B). Most active protein kinases contain an alanine here, often as part of a V**A**IK motif (Table S5), though other small hydrophobic residues (V/I/L/M) are also acceptable (valine is seen in active VRKs, Figure 2A). However, 16 pseudokinases (33%) have unusual substitutions, and 10 of these are serine or threonine residues (Table S5), which would be capable of forming a similar interaction to that of S201 in VRK3 (Figure 2B, see above). Unlike the G-loop motif, no active kinase violates the expanded consensus motif for this position. Thus, whereas this marker is less sensitive, it appears to be highly specific.

Using these additional motifs, and unconventional active kinases (WNKs, Titin, and CASK) as a guideline, we looked for pseudokinase sequences that have similarly moderate deficits, and thus could possibly retain cryptic activity. This survey revealed 12 pseudokinases that had an intact G-loop motif (or one that was plausibly compatible with activity, as in the WNKs) and only one other deficit in the four other motifs, as seen in all the unconventional kinases (Table 1). All other pseudokinases had highly degraded G-loop motifs and/or more degradation of other key motifs (Table S5), further supporting predictions of their inactivity.

### Key Catalytic Motifs in VRK3 Retain Structural, but Not Functional, Residues

In addition to loss of ATP binding, the distinctive kinase catalytic motifs are also degraded in VRK3 (Figures 2A and 2C). The aspartate in the catalytic loop hr**D**xkxxN (D166^VRK2^), which orients the hydroxyl group of the substrate residue (Madhusudan et al., 2002) and is essential for catalytic activity (Gibbs and Zoller, 1991), is replaced by an asparagine in human VRK3 (N306) and in ∼50% of VRK3 orthologs. VRK3 has also completely lost the neighboring conserved lysine (K168^VRK2^, T308^VRK3^), which forms key interactions in the active site, and is thought to be important in the catalytic mechanism (Bossemeyer et al., 1993; Madhusudan et al., 2002). Finally, the aspartate in the metal-binding **D**fG motif (D186^VRK2^), conserved in virtually all active kinases, is lost in VRK3, indicating limitations in ATP stabilization and phosphotransfer.

By contrast, residues that also play a structural role in stabilizing the kinase conformation, such as the K203-E214^VRK3^ ion pair (K72-E91^PKA^), have been retained. Likewise, both H304 and N311^VRK3^ of the **h**rDxkxx**N** motif are retained; these stabilize the active site conformation by hydrogen bonding to the backbones of A325 and N306^VRK3^, respectively (Figures 2A and 2C). As a result, the active site conformations of VRK3 and VRK2 are very similar, despite the inactivity of VRK3 (Figure 2C).

### The Activation Segment of VRK3 Is Distinct from that of Active VRKs

**Figure 4:**
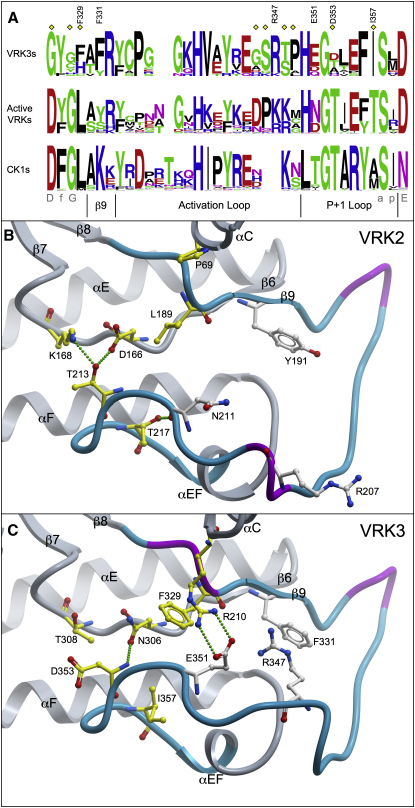
Changes in the VRK3 Activation Segment (A) Sequence conservation logos for the full activation segments of VRK3s, active VRKs, and the CK1 family, based on appropriate subsets of a curated sequence/structure alignment of diverse homologs (see Experimental Procedures), labeled with same conventions as Figure 2A. Subsections of the activation segment are shown below the motifs. (B) The activation segment of VRK2, shown in cyan, with 3–10 helices in violet and structure outside of the activation segment in gray. Residues are shown with same conventions as Figures 2B and 2C. Portions of the structure are not shown to improve clarity. (C) VRK3 activation segment, labeled as in panel B.

The activation segment of protein kinases runs from the DfG motif (DyG in VRKs) to an APE motif (SxN in most CK1 group kinases, SxD in VRKs) (Nolen et al., 2004). Between these two motifs are an activation loop, which activates many kinases when phosphorylated, and a p+1 loop, which is important in substrate binding (Figure 4A). The activation segments of VRK2 and VRK3 are well defined and adopt a surprisingly similar conformation, though there are changes in VRK3 that might result from its catalytic inactivity (Figures 4B and 4C).

VRK3 retains the central tyrosine in the DyG motif, and partially conserves the glycine, but replaces the essential aspartate with a conserved G326^VRK3^ (Figure 4A). By contrast, it retains the SxD motif at the end of the loop, probably due to its structural role in anchoring the loop and stabilizing the C-terminal subunit (Nolen et al., 2004; Scheeff and Bourne, 2005). The sequence of the rest of the VRK3 activation segment is also largely conserved and similar to that of active VRKs, correlating with the observed structural similarity and suggesting a retained functional role.

However, toward the end of the activation loop, where all VRKs have an insert relative to CK1, the sequences and structures of VRK2 and VRK3 diverge (Figure 4). In VRK2, residues 206–208 form a short 3-10 helix, with the charged R207 facing out to the solvent, opening a groove through the middle of the activation loop that might participate in substrate binding (Figure 4B). In VRK3, this helix is replaced by a kink, in which the equivalent basic residue (R347) flips inward, partly filling in the middle of the loop region and forming a cation-π interaction with a conserved aromatic residue at F331^VRK3^. This VRK3-specific change might further stabilize the activation segment, perhaps simultaneously occluding a region that would otherwise bind substrate in an active VRK. Second, a cluster of 3 residues (R210, F329, E351^VRK3^) come together to directly block part of the interaction surface of the p+1 loop and so occlude part of the potential substrate binding region immediately adjacent to (what would otherwise be) the catalytic center of the enzyme. The interactions between these residues also form a bridge that links the N-terminal and the C-terminal lobes, likely further stabilizing VRK3 in a closed pseudo-active conformation (Figure 4C). Finally, all active VRKs conserve two threonines (T213, T217^VRK2^) in the p+1 loop that are lost in VRK3 (Figure 4A). T213 is found in most serine/threonine kinases, anchoring the loop and organizing the active site via hydrogen bonds to D166 and K168^VRK2^ in the hr**D**x**k**xxN catalytic loop motif (Nolen et al., 2004) (Figure 4B). Neither of these interacting residues is conserved in VRK3 (Figure 4C). T217^VRK2^ forms a hydrogen bond to the backbone of N211^VRK2^ to help stabilize the position of the activation segment (Figure 4B), and thus its loss in VRK3 may reflect the change in conformation of this segment. Although the structural differences seen between VRK3 and VRK2 could simply represent alternate conformations that occur in both structures, this is unlikely: the structural changes coincide with distinct sequence changes in VRK3 that violate the evolutionary constraints in the active VRKs (Figure 4), indicating that they are the likely cause of the structural reorganization. These changes might eliminate substrate binding in VRK3, though relatively little is known about substrate interaction by CK1 group kinases (Longenecker et al., 1996; Nolen et al., 2004; Xu et al., 1995), and it is possible that VRK3 still uses this modified region in interactions with other proteins.

### VRK3 Displays an Inverted Pattern of Surface Conservation, Suggesting that the Back of the Enzyme Forms Key Functional Interactions

**Figure 5:**
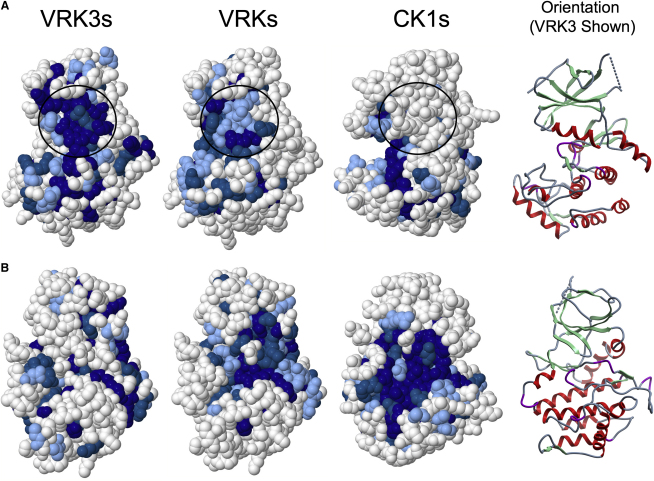
Surface Conservation Patterns for VRK3s Only, All VRKs, and CK1s (A) View of the face of the molecules opposing the active site. The top three of nine conservation bins from ConSurf (see Experimental Procedures) are shown in corresponding shades of blue (darkest blue indicates the most conserved). Results for all VRKs are mapped onto the VRK3 structure, but are valid for all family members. Results for CK1s are mapped onto the CK1 structure. Conservation scoring is scaled to each sequence grouping, and is not directly comparable between groups. The unique conserved patch present in VRKs, but not CK1s, is circled. (B) Molecules from panel A are rotated ∼120° such that the active site and substrate binding region is facing forward.

**Figure 6:**
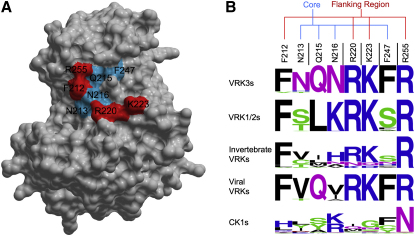
Putative Binding Site on the Back of VRK3 (A) Key residues of the putative binding patch, mapped to a surface view of VRK3. Red residues are conserved in all VRKs, and blue are conserved but with distinctive patterns in VRK3 versus VRK1/2. (B) Motif logos of site in different VRKs and the CK1 family. Sequence is a catenation of residues distributed within the sequence alignment: residues in the VRK3 structure are numbered above the alignment, and black bars indicate break points where intervening sequence was removed. The two subsets of conserved residues are indicated above the motifs.

The lack of ATP binding and catalytic activity, coupled with the surprisingly conserved fold of VRK3, suggested that the VRK3 structure might be maintained to provide a binding surface for other proteins, a known function of many pseudokinases (Boudeau et al., 2006). VRK3 has been shown to bind and activate the phosphatase VHR (Kang and Kim, 2006), so we sought to investigate the structural basis of this (and other possible) interactions. Using curated homologous sequences from a wide range of organisms, we searched for selectively conserved patches of surface residues. We compared VRK3 orthologs with active VRKs and with CK1 family kinases, mapping residue conservation to the relevant structures using ConSurf (Landau et al., 2005). This revealed a large patch of highly conserved residues on the face of VRK3 opposite the active site, from αC, β4, and β5, and the activation segment (Figure 5A). This patch is in contrast to the active site region of VRK3, which has a poorly conserved surface (Figure 5B), consistent with an inactive enzyme. A subset of the VRK3 patch (with residues from αC, β4, and β5) is conserved in all VRKs, but not in the CK1 family, suggesting that the patch mediates a VRK-specific function that has been further adapted in VRK3 (Figures 5A and 6). Within this smaller patch, we found a highly conserved flanking region consisting of 4 pan-VRK residues, of which 3 are basic, surrounding a core that includes 4 residues that are conserved but distinct between VRK3 and VRK1/2 (Figure 6). This pattern suggested that the flanking region might present a general protein interaction site, whereas the core provides binding specificity between VRK3 and VRK1/2. Examination of the core revealed that, remarkably, viral VRKs display a pattern that is most similar to VRK3, suggesting that viral VRKs might have evolved to form a similar interaction to VRK3, despite being active enzymes (Figure 6B).

## Discussion

The twin structures of VRK2 and VRK3 provide the first insights into the structural consequences of loss of catalytic activity in a pseudokinase. They also enable a deeper understanding of the remaining regulatory functions that have been proposed for many pseudokinases (Boudeau et al., 2006). Surprisingly, the overall fold is highly conserved between the two structures, contrary to expectations from sequence alignment that the VRK3 structure would be degraded. This strong constraint on structure, relative to loss of overall sequence constraints, is further seen in the key kinase motifs, where residues that have structural roles are largely conserved whereas neighboring catalytic residues are lost. Along with the rigidity of the activation segment and G-loop, these observations suggest that VRK3 might have been retained as a scaffold or regulatory binding partner.

One clue to the structural conservation of VRK3 is the conserved patch opposite the active site. Several structural elements contribute to the patch, and are likely to be constrained by this function. The patch was not obvious from the linear sequence alone, and required a combination of the crystal structure and prediction of orthologous sequences to be identified. The observed conservation pattern (Figure 6) suggests that VRK3 uses the patch for a distinct function that cannot be replicated by another VRK, perhaps for its unique capability among vertebrate VRKs to interact with and activate VHR (Kang and Kim, 2006, 2008). Because VHR and VRK3 are both vertebrate specific, and all VRK3 orthologs are predicted to be inactive, it is likely that VRK3 regulation of VHR emerged early in vertebrate evolution from a pre-existing interaction site, allowing VRK3 to be retained even in the absence of any catalytic function. The patch motif in viral VRKs is most similar to that of VRK3 (Figure 6), raising the intriguing possibility that viral VRKs, though active kinases, might have evolved to bind the same partner as VRK3, and perhaps interfere with VRK3-mediated regulation.

VRK3 might bind other partners, perhaps acting as a scaffold to integrate multiple signals in regulation of VHR. This sort of scaffolding role has been seen in other pseudokinases (Boudeau et al., 2006) and in the noncatalytic C terminus of VRK2. Additional interactions could provide an alternate explanation for the observed patch, along with the other smaller regions of conservation seen on the surface of VRK3 (Figure 5), and several studies have indicated that such interactions occur. Incubation of VRK3 with HT22 cell lysate results in phosphorylation of VRK3 and copurification of a kinase activity, suggesting that it might bind to and be phosphorylated by an active kinase (Kang and Kim, 2006). Disruption of the K203-E214 ion pair (K72-E91^PKA^) abolished this phosphorylation, confirming that the structural integrity of VRK3 is required for this interaction. Erk, VHR, and VRK3 coimmunoprecipitate as a ternary complex, and it has been suggested that VRK3 provides an Erk interaction site for VHR, because VHR lacks the MAPK binding domain found in most other MAPK phosphatases (Kang and Kim, 2008). A recent report shows that the small nuclear GTPase, Ran, is another VRK3 binding partner and also binds and catalytically inhibits VRK1 and VRK2 (Sanz-Garcia et al., 2008).

The VRK3 structure also highlights the importance of the G-loop and β3 motif in ATP binding and kinase activity. Mutations to the G-loop motif have a drastic effect on the catalytic efficiency in active kinases (Grant et al., 1998). The G-loop motif degeneration in VRK3 corroborates the loss of ATP binding shown by thermal denaturation assay. Loss of the motif correlates well with loss of catalytic residues in other pseudokinases (Manning et al., 2002b), and helps to refine these predictions (Table S5). For example, both Titin and CASK were predicted to be pseudokinases based on loss of the **D**FG aspartate, but both retain the G-loop and β3 motifs, and this correlates with their observed catalytic activities (Mukherjee et al., 2008; Takano-Ohmuro et al., 1992) (Table 1). Conversely, Ryk retains the 3 main catalytic residues but lacks the consensus G-loop and β3 motifs, and has convincingly been shown to be catalytically inactive (Katso et al., 1999) (Table 1). However, these additional motifs are not completely predictive: a few active kinases also lack the consensus G-loop motif (Table S5), and some pseudokinases (such as STRADs and guanylyl cyclases) bind ATP despite deficits in one of the two motifs (Table 1), and might require it for (noncatalytic) function (Boudeau et al., 2004; Jaleel et al., 2006). By combining all motif data, we detected 12 pseudokinases that would the most likely to be cryptically active, because of mild modifications to the G-loop motif, coupled to loss of only one of the other key motifs (Table 1). However, there are reasons to believe that activity in many of these proteins is unlikely: seven have mutations to the aspartate residue of the hr**D** motif, a position that is thought to be essential to activity (Gibbs and Zoller, 1991), and many have been found to be inactive in experiments (Table 1). Still, there are examples of loss of the hrD motif in distantly related microbial kinases (Kannan et al., 2007) and, as with CASK (Mukherjee et al., 2008), it is possible that some pseudokinases could be active only in unusual conditions. Thus, although 49 pseudokinase domains are predicted to be inactive like VRK3, a few might hold surprises.

It remains to be seen how predictive the structural features seen in VRK3 will be for other pseudokinases. Unlike active kinases, which are constrained in evolution by a shared catalytic activity, each pseudokinase faces a distinct set of evolutionary pressures dictated by its unique remaining functions. Indeed, this pattern is reflected in their varied patterns of motif degradation (Table S5). However, the strong structural similarity between VRK3 and VRK2 suggests that the ∼25 human pseudokinases with severe sequence degeneration (Manning et al., 2002b) might still produce a highly conserved fold. Such fold conservation makes evolutionary sense: a pseudokinase ancestor would almost certainly “learn” its noncatalytic interactions while still an active kinase. Once catalytic activity was lost, maintaining these interactions could require the pseudokinase to mimic the surface of its active ancestor. Thus, the result would likely be a pseudokinase with a conserved structure, as seen in VRK3. Because other pseudokinases also participate in multiple protein-protein interactions (Boudeau et al., 2006), it is likely that most will look very much like active kinases, despite their catalytic deficits.

Addressing the mystery of VRK3 function benefited from a close integration of structural analysis and comparative genomics. The complexity of the evolutionary pressures on pseudokinases argues for the use of such a combined approach, because it allows observed structural features to be placed in evolutionary context. In VRK3, the combination was able to distinguish motif residues involved in functional versus structural roles, identify conservation patches built from multiple distinct sequence regions, and highlight the remaining constraints on this important “dead” kinase. We anticipate that, as here, the fusion of genomics and structural genomics will soon provide more insight into the retained features and functions of other pseudokinase domains.

## Experimental Procedures

### Cloning of Structural Sequences

Catalytic domain residues were amplified from cDNA provided by FivePrime. VRK1 (residues 1–365), VRK2 (15–335), and VRK3 (14–-472) were cloned into the vector, pNIC-SGC, by ligation-independent cloning (Stols et al., 2002). The vector includes a TEV-cleavable (^∗^) N-terminal His_6_ tag (MHHHHHHSSGVDLGTENLYFQ^∗^SM). The TEV cleavage site is present in the structure of VRK3 (positions 137–145) and generates an α helix from positions 138–147 (omitted from all figures).

### Expression and Purification

Transformed BL21(DE3) cells were grown in Luria-Bertani medium containing 50 μg/ml kanamycin. Protein expression was induced at an OD_600_ of 0.8 using 1 mM isopropyl-thio-galactopyranoside (IPTG) at 20°C for 12 hr. Cells expressing VRK2 or VRK3 were lysed in 50 mM HEPES (pH 7.5), 500 mM NaCl, 1 mM PMSF, and 0.5 mM TCEP using an EmulsiFlex high-pressure homogenizer. After centrifugation, the supernatant was loaded onto a Nickel-Sepharose column equilibrated in 30 ml binding buffer (50 mM HEPES [pH 7.5], 500 mM NaCl, 5 mM imidazole, 0.5 mM TCEP, and 5% glycerol). The column was washed with 3 × 10 ml wash buffer (loading buffer with 30 mM Imidazole). Proteins were eluted by an imidazole step gradient and applied to a Superdex 200 16/60 gel-filtration column equilibrated in 50 mM HEPES (pH 7.5), 150 mM NaCl, and 5 mM DTT.

### Crystallization

Crystallization was performed using sitting drops, mixing protein (8–10 mg/ml) and well solutions in 2:1, 1:1 and 1:2 ratios. VRK3 apocrystals were obtained using a well solution containing 0.1 M HEPES (pH 7.5) and 2 M ammonium formate at 4°C. VRK2 was crystallized using 0.1 M Na(succ) and 15% PEG 3350 at 20°C.

### Data Collection and Processing

Crystals were cryoprotected using the well solution supplemented with an additional 20% ethylene glycol and flash frozen in liquid nitrogen. Diffraction data were collected on the PXII beamline at the Swiss Light Source at 1.00 and 1.03 Å wavelengths for VRK2 and VRK3, respectively. Data were processed using XDS. Multiple segments were scaled together using XSCALE (Kabsch, 2001).

### Structure Solution, Model Building, and Refinement

VRK3 was solved using an ensemble of CK1 structures (Protein Data Bank [PDB] codes 2CMW, 2C47, 2IZR, 2IZS, 2IZT, 2IZU, 2CHL, 1CKI, and 1CKJ). NCS-averaging was used to improve initial maps, and ARP/wARP was used to produce an initial trace. The resulting map was clear enough to allow manual model completion. Alternating cycles of refinement using REFMAC5 and manual editing in Coot (Emsley and Cowtan, 2004) were performed until convergence.VRK2 was solved using VRK3 as a search model. The initial map was improved using ARP/wARP, followed by NCS-averaging, after which the structure could be autobuilt using ARP/wARP and refined using alternating cycles of REFMAC5 and Coot.

### Structure Alignment and Analysis

All structural coordinates were collected from the PDB, and processed through MolProbity (Davis et al., 2007) to add optimized hydrogen atoms. All Asn/Gln/His residue flips suggested by MolProbity were also applied. Structures were aligned and superimposed using DaliLite (Holm and Park, 2000). Published structures included CK1 (1CSN) (Xu et al., 1995), Sky1p (1Q97) (Nolen et al., 2003), and PKA (1CDK) (Bossemeyer et al., 1993). In all cases, chain A from the PDB file was used, with the exception of VRK3, in which chain B was used. For the electrostatic models (Figure 3), the missing G-loop in VRK2 (residues G36-G41) was modeled in Coot (Emsley and Cowtan, 2004) and further optimized in ICM (Molsoft L.L.C., La Jolla, CA). The electrostatic surfaces for VRK3 and VRK2 were then calculated using ICM.

### Sequence Prediction and Alignment

To measure the evolutionary constraints on vertebrate VRKs, we predicted VRK sequences for a wide range of vertebrates: five fishes, six mammals, chicken, and frog, and from a range of invertebrate metazoans and viruses, using Gene Detective (Time Logic, Carlsbad, CA) and GeneWise (Birney et al., 2004) predictions, and Ensembl (Hubbard et al., 2007) and GenBank (Benson et al., 2008) protein and EST sequences. Predicted sequences were manually curated. VRK and CK1 sequences were aligned using muscle (Edgar, 2004), followed by manual editing, with the help of structure-based sequence alignments from DaliLite (Holm and Park, 2000) (Figure S1). All sequences are available at http://kinase.com.

### Surface Conservation and Motif Analysis

Surface conservation was measured using the ConSurf server, with the default (Bayesian) method (Landau et al., 2005). The appropriate subsets of the above curated alignment were used as the sequence inputs to the ConSurf algorithm for each family. Logos were generated with the WebLogo server (Crooks et al., 2004).

### Thermal Stability Assay for ATP, Inhibitor, and Cation Binding

Proteins were assayed for shifts in melting temperature caused by presence of MgCl_2_ or MnCl_2_ (2.5 mM), ATP (5 mM), or ATP-mimetic kinase inhibitors (10 mM). Thermal melting experiments were done using a Mx3005p RT-PCR machine (Stratagene). The purified VRK1, VRK2, and VRK3 proteins were buffered in 10 mM HEPES (pH 7.5) and 150 mM NaCl and assayed in a 96-well plate at a final concentration of 5 mM in 20 ml volume. SYPRO-Orange (Molecular Probes) was added as a fluorescence probe at a dilution of 1 in 1000. Excitation and emission filters for the SYPRO-Orange dye were set to 465 and 590 nm, respectively. The temperature was raised with a step of 1°C per minute from 25°C to 96°C and fluorescence readings were taken at each interval. The temperature dependence of the fluorescence during the protein denaturation process was approximated by the equation

$$y\left(T\right)=y_{F}+\frac{y_{U}-y_{F}}{1+e^{\Delta uG_{\left(T\right)}/RT}}$$

where ΔuG is the difference in unfolding free energy between the folded and unfolded state, R is the gas constant and y_F_ and y_U_ are the fluorescence intensity of the probe in the presence of completely folded and unfolded protein, respectively (Matulis et al., 2005). The baselines of the denatured and native state were approximated by a linear fit. The observed temperature shifts, Δ*T*_m_^obs^, for each inhibitor were recorded as the difference between the transition midpoints of sample and reference wells containing protein without inhibitor in the same plate and determined by nonlinear least-squares fit. Each Tm value given (Figure 3D) is an average of three experimental measurements. Values given for native Tm in the text are an average of five experimental measurements.

## Figures and Tables

**Table 1:** Key Motifs from Human Sequences of Unconventional Active Kinases, Selected Pseudokinases, and Pseudokinases with Moderate Deficits

Kinase	G-Loop	G-Loop Status	β3	hr**D**	**D**fG	Notes
Unconventional Active Kinases						

CASK	**G**K**G**PF**S**	I	F**A**V**K**	HR**D**	GFG	Active, compensation for missing **D**fG is unknown (Mukherjee et al., 2008)
WNK1	**G**R**G**SFK	P	V**A**WC	HR**D**	**D**LG	Active, K in G-loop compensates for missing K in β3 (Min et al., 2004)
Titin	**G**R**G**EF**G**	I	Y**M**A**K**	HF**D**	EFG	Active, structure shows that E of EFG functionally replaces D (Mayans et al., 1998)

Selected Pseudokinases						

VRK3	TRDNQ**G**	D	FSLK	HGN	GFG	Experimentally inactive (Nichols and Traktman, 2004), cannot bind ATP
HSER (GC-C)	RRDTIQ^a^	D	V**I**L**K**	HGR	**D**FG	Binds ATP despite highly degraded G-loop motif, experimentally inactive (Jaleel et al., 2006)
STRAD (STLK5)	**G**K**G**FED	P	VTVR	HRS	GLR	Binds ATP despite moderate degradation in G-loop and substitution in β3, experimentally inactive (Boudeau et al., 2004)
ILK	NENHS**G**	D	IVV**K**	RHA	**D**VK	Experimentally inactive (Boudeau et al., 2006)
EphB6	**G**T**G**SF**G**	I	VAIQ	HRS	RLG	Experimentally inactive (Gurniak and Berg, 1996)
HER3 (ErbB3)	**G**S**G**VF**G**	I	VCI**K**	HRN	**D**FG	Experimentally inactive, reversion of HRN to HRD fails to reactivate (Prigent and Gullick, 1994)

Pseudokinases with Moderate Deficits						

RYK	QE**G**TF**G**	P	AFV**K**	HK**D**	**D**NA	Experimentally inactive, reversion of DNA to DFG reactivates (Katso et al., 1999)
KSR1	**G**Q**G**RW**G**	I	V**A**IR	HK**D**	**D**FG	Experimentally inactive (Kolch, 2005; Morrison, 2001)
KSR2	**G**K**G**RF**G**	I	V**A**IR	HK**D**	**D**FG	Experimentally inactive (Kolch, 2005; Morrison, 2001)
CCK4	**G**KSEF**G**	P	V**L**V**K**	HK**D**	ALG	Experimentally inactive, repair of altered **D**fG motif in *Hydra* ortholog fails to reactivate (Kroiher et al., 2001)
SuRTK106	CS**G**SC**G**	P	V**I**L**K**	HG**D**	GLG	
IRAK3 (IRAK-M)	**G**E**G**EIF	P	Y**A**V**K**	CGS	**D**FA	Experimentally inactive (Wesche et al., 1999)
JAK1 Dom2	**G**R**G**TRT	P	V**I**L**K**	HGN	**D**PG	
JAK2 Dom2	**G**Q**G**TFT	P	V**L**L**K**	HGN	**D**PG	
JAK3 Dom2	**G**H**G**SFT	P	V**L**L**K**	HGN	**D**PG	
TYK2 Dom2	**G**Q**G**TRT	P	V**V**L**K**	HGN	**D**PG	
SgK071	NP**G**AL**G**	P	H**V**I**K**	HRN	**D**LS	
PSKH2	**G**T**G**SF**S**	I	F**A**I**K**	HRN	**D**FG	

Key residues are in bold when conserved and underlined when not conserved. G-loop status scores conservation of GxGxxG motif: I indicates intact, conforms to consensus; p, plausible, with conservative changes; D, degraded, severe changes. Pseudokinases with moderate deficits have a G-loop motif that is either intact or plausible, and no more than one other defect in other motifs. See Table S5 for data on all pseudokinases.

a Motif is a best estimate, because we were unable to align this region to active kinases with confidence.

## Accession Numbers

The structure and corresponding structure factors for VRK2 and VRK3 were deposited in the Protein Data Bank under accession numbers 2V62 and 2JII, respectively (Table S1).

## Supplemental Data

Document S1. Four Tables and One Figure

Table S5. Summary of Motif Information for Active and Predicted Pseudokinases
