# Supplementary material for: Structure of the Pseudokinase VRK3 Reveals a Degraded Catalytic Site, a Highly Conserved Kinase Fold, and a Putative Regulatory Binding Site
Source: Structure. 2009 Jan 14;17(1):128–38. doi: 10.1016/j.str.2008.10.018 (PMC2639636; doi:10.1016/j.str.2008.10.018)
Supplement: Document S1. Four Tables and One Figure [file mmc1.pdf]

Supplemental Data

Structure of the Pseudokinase VRK3 Reveals a Degraded Catalytic Site, a Highly Conserved Kinase Fold, and a Putative Regulatory Binding Site

Eric D. Scheeff, Jeyanthy Eswaran, Gabor Bunkoczi, Stefan Knapp,and Gerard Manning

Table S1. Crystallographic Data and Refinement Statistics

**Data Collection**	**VRK2**	**VRK3**
Space group	$P2_12_12$	$P2_1$
Cell dimensions [Å and °]	a=70.65, b=157.53, c=56.29	a=91.26, b=54.42, c=91.82, β=91.2
Resolution*	1.7 (1.7-1.8)	2.0 (2.0-2.1)
Unique observations*	67759 (10022)	60969 (8014)
Completeness* [%]	96.9 (93.3)	99.2 (95.4)
Redundancy*	5.8 (5.5)	3.6 (2.6)
R_{merge}*	0.0562 (0.5859)	0.0648 (0.4167)
I / σI*	15.3 (2.3)	12.5 (2.2)
**Refinement**		
Reflections (R_{free} set)	64302 (3413)	57853 (3092)
R_{work} / R_{free}	0.201 / 0.236	0.178 / 0.223
Atoms (P/L/W)#	4828 / 21 / 334	5143 / 20 / 514
B-factors (P/L/W)# [Å^2]	34.1 / 50.0 / 38.5	34.9 / 49.0 / 40.8
RMSD bonds [Å]<br>RMSD [°]	0.015<br>1.483	0.011<br>1.265
**Ramachandran**<br>favorable [%]<br>allowed [%]	 99.6<br>0.4	 100.0<br>0.0

Table S2. Superpositions between VRK2 and VRK3 and Other Representative Protein Kinases, as Aligned by DaliLite (Holm and Park, 2000)

**Structure** (PDB ID: Chain)	**VRK3** (2JII:B)	**VRK2** (2V62:A)	**CK1** (1CSN:A)	**PKA** (1CDK:A)
**VRK2** (2V62:A)	2.3 (291,38,35.4)			
**CK1** (1CSN:A)	2.1 (271,23,30.3)	2.4 (266,27,29.2)		
**PKA** (1CDK:A)	3.1 (257,16,22.2)	3.1 (241,18,20)	2.8 (258,19,23.9)	
**Sky1p** (1Q97:A)	3.0 (272,15,23.6)	3.1 (262,19,21)	2.6 (250,20,22.9)	2.7 (251,26,24)

RMSD is given in Å, followed by additional information on the structural comparison in parentheses: number of aligned positions, % sequence identity, and DaliLite Z-score. Comparisons between VRK3 and VRK2 and paralogs are highlighted in gray.

Table S3. Inhibitor Screening Results for VRK1

Compound name	Chemical structure	VRK1 Tm Shift [ºC]
3-(2-Chloro-3-indolylmethylene)-1,3-dihydroindol-2-one	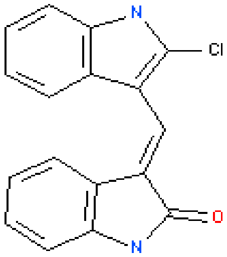	4
ASC67	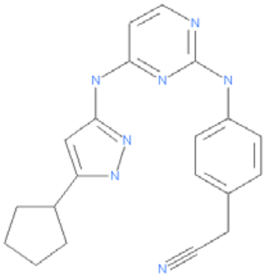	4.3
ASC24	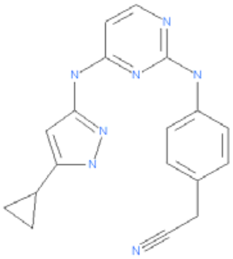	4.5
Oxindole I; 3-(1H-Pyrrol-2-ylmethylene)-1,3-dihydroindol-2-one	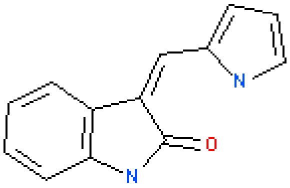	3.5
5-Iodo-3-[(3,5-dibromo-4-hydroxyphenyl)methylene]-2-indolinone	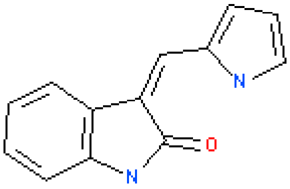	3.5

Table S4. Inhibitor Screening Results for VRK2

Compound name	Chemical structure	VRK2 Tm Shift [ºC]
ASC67	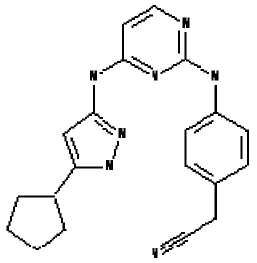	4.5
3-(2-Chloro-3-indolylmethylene)-1,3-dihydroindol-2-one	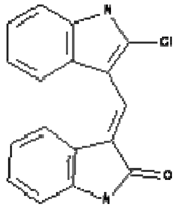	4
ASC24	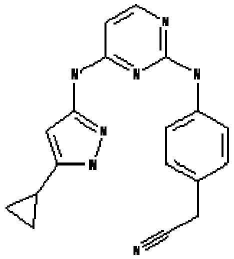	4
"Oxindole I; 3-(1H-Pyrrol-2-ylmethylene)-1,3-dihydroindol-2-one"	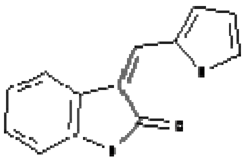	3.5
ASC29	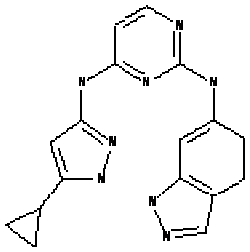	3.5

Figure S1. Curated Alignment of VRKs and CK1s

Key sections discussed in text are highlighted.

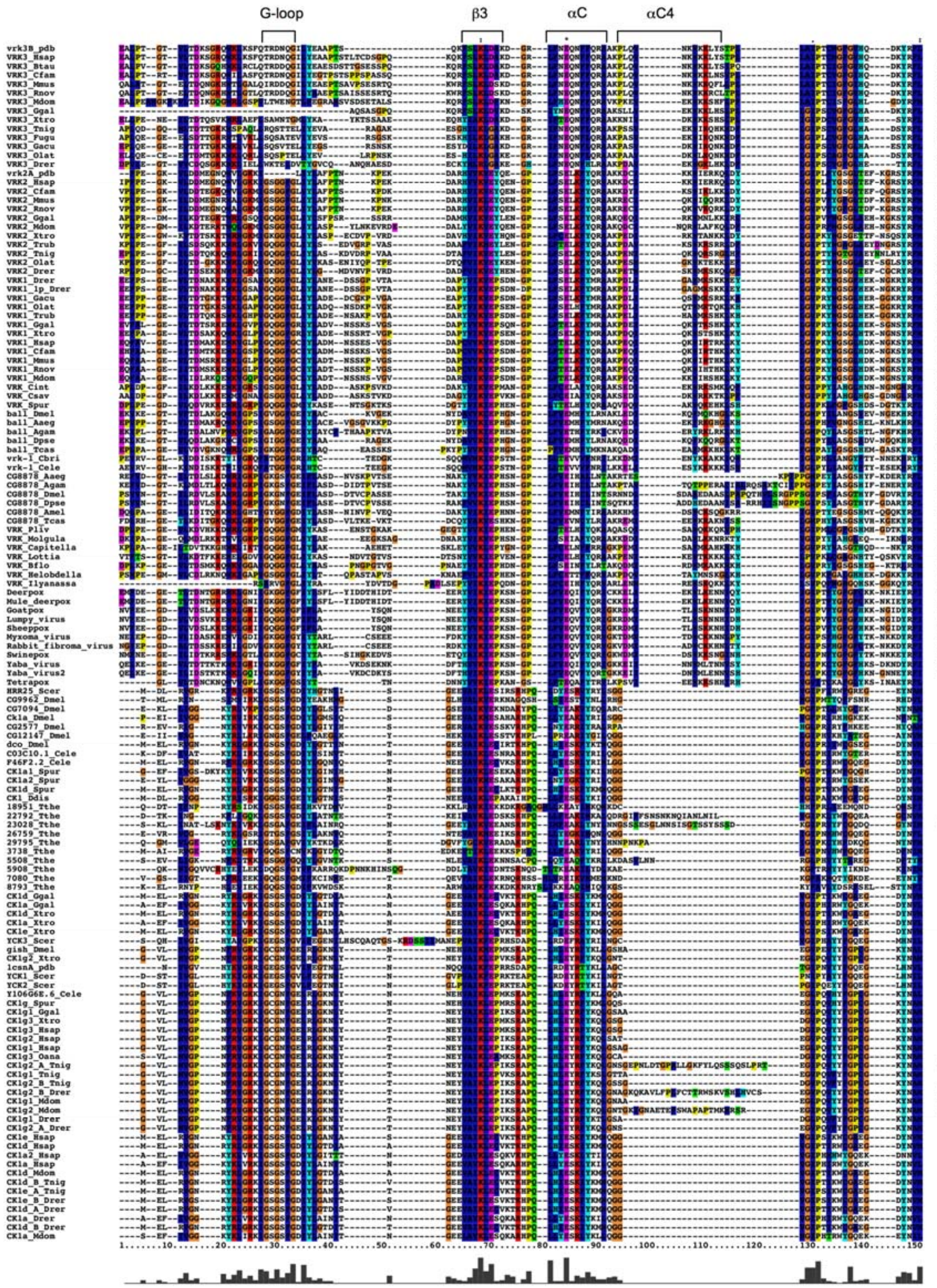

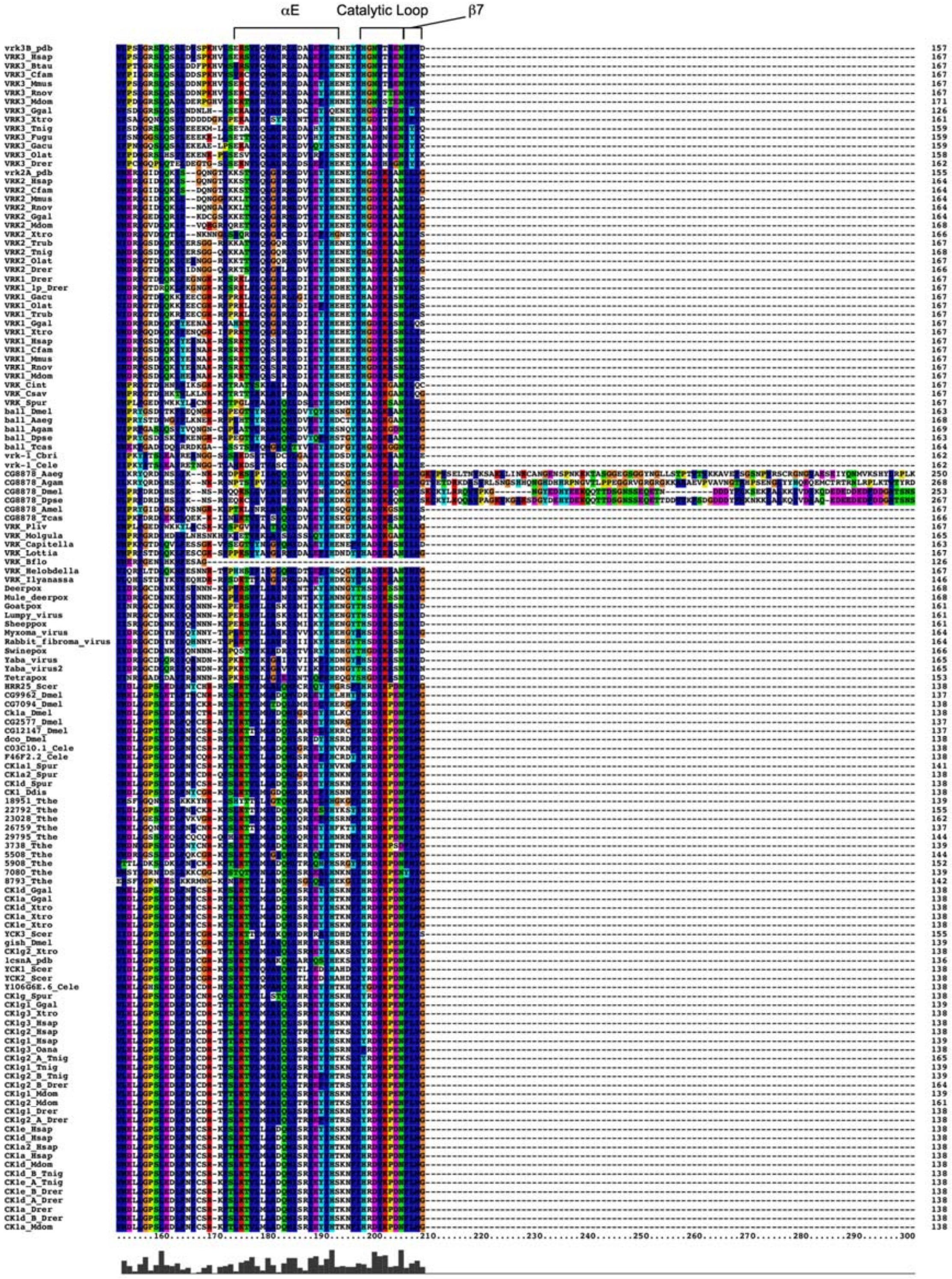
αE
Catalytic Loop
β7

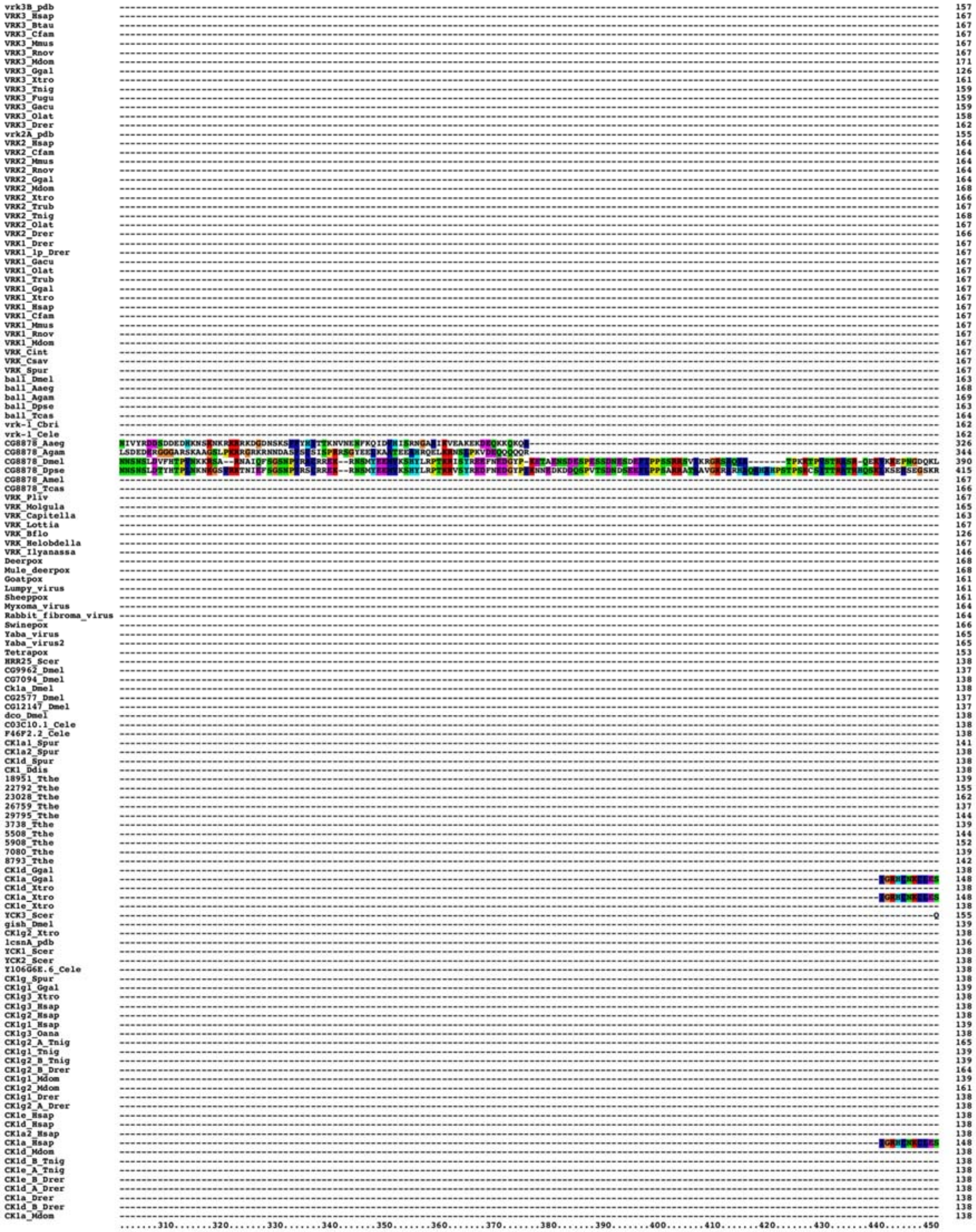

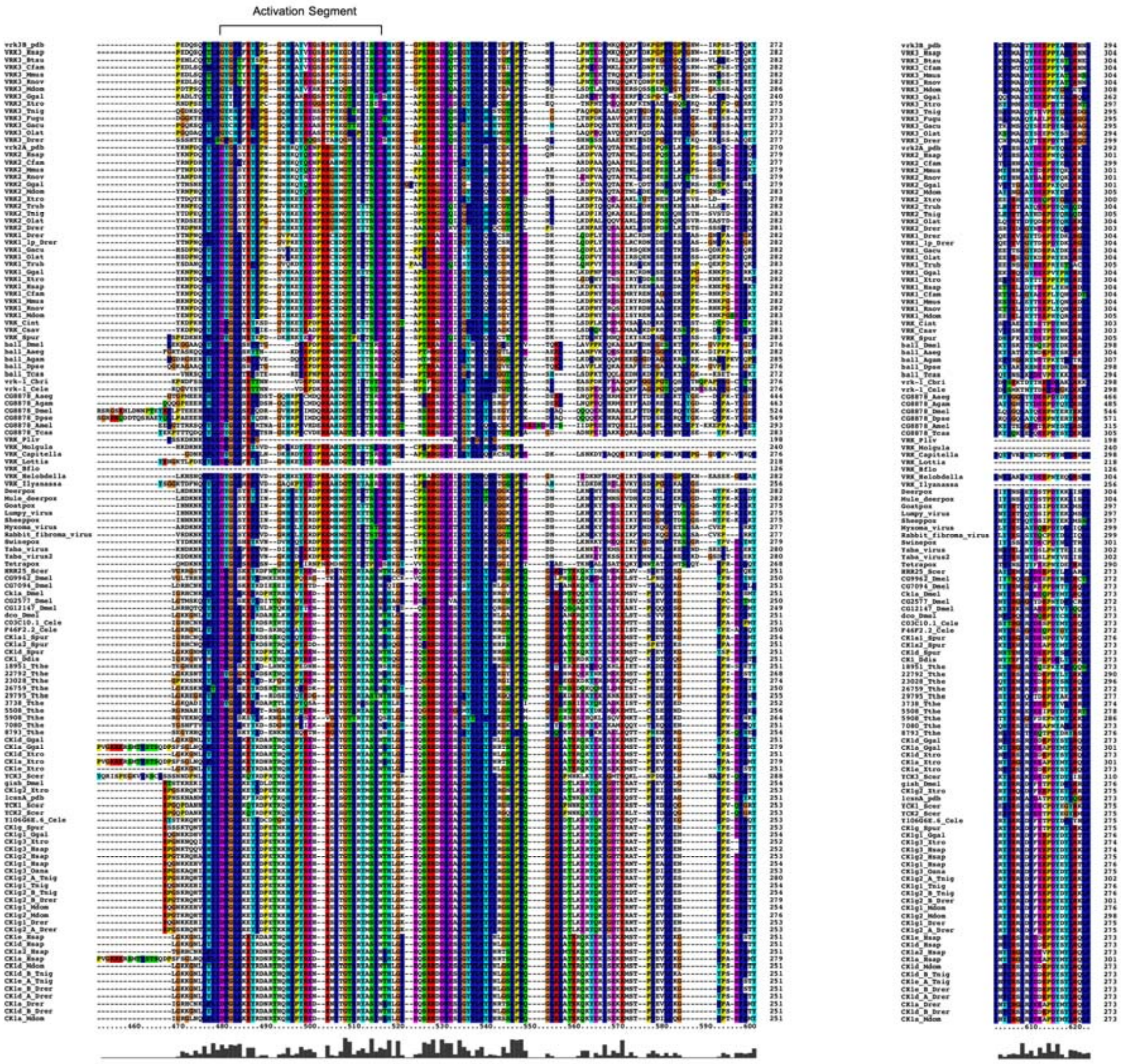
Activation Segment
